# eQTLs are key players in the integration of genomic and transcriptomic data for phenotype prediction

**DOI:** 10.1186/s12864-022-08690-7

**Published:** 2022-06-28

**Authors:** Abdou Rahmane Wade, Harold Duruflé, Leopoldo Sanchez, Vincent Segura

**Affiliations:** 1 INRAE, ONF, BioForA, UMR 0588, F-45075 Orleans, France; 2 UMR AGAP Institut, Univ Montpellier, CIRAD, INRAE, Institut Agro, F-34398 Montpellier, France

**Keywords:** Genomic Prediction, Omics, Multi-omics integration, eQTL, *Populus nigra*

## Abstract

**Background:**

Multi-omics represent a promising link between phenotypes and genome variation. Few studies yet address their integration to understand genetic architecture and improve predictability.

**Results:**

Our study used 241 poplar genotypes, phenotyped in two common gardens, with xylem and cambium RNA sequenced at one site, yielding large phenotypic, genomic (SNP), and transcriptomic datasets. Prediction models for each trait were built separately for SNPs and transcripts, and compared to a third model integrated by concatenation of both omics. The advantage of integration varied across traits and, to understand such differences, an eQTL analysis was performed to characterize the interplay between the genome and transcriptome and classify the predicting features into *cis* or *trans* relationships. A strong, significant negative correlation was found between the change in predictability and the change in predictor ranking for *trans* eQTLs for traits evaluated in the site of transcriptomic sampling.

**Conclusions:**

Consequently, beneficial integration happens when the redundancy of predictors is decreased, likely leaving the stage to other less prominent but complementary predictors. An additional gene ontology (GO) enrichment analysis appeared to corroborate such statistical output. To our knowledge, this is a novel finding delineating a promising method to explore data integration.

**Supplementary Information:**

The online version contains supplementary material available at 10.1186/s12864-022-08690-7.

## Background

Genomic prediction, the prediction of phenotypes with genome-wide polymorphisms, has become a key tool for plant and animal breeders. This approach relies on statistical modelling to infer the effect of genomic variants, with many different modelling alternatives proposed in the literature [1, 2]. These models are mostly devised to predict the additive and transmissible contribution to individual genetic values, although dominance and epistatic interactions can also be accounted for [2]. Despite their success in identifying relevant effects and predicting phenotypes accurately, even in their most complex formulations, these models do not capture per se the genetic architecture of complex traits [3]. Beyond the statistics, it is the use of biological and functional information from the different organizational layers between the raw sequence and the organismal phenotype that will likely provide the required insights into genetic architectures. Layers such as DNA methylation (Epigenome), transcripts (Transcriptome), proteins (Proteome) or metabolites (Metabolome), are nowadays becoming increasingly accessible for many species, opening prospects for a better understanding of the genetic architecture of complex traits [4–6].

In order to simultaneously account for these different layers of data in phenotype prediction, several integration approaches have been proposed [7]. Among these, the most frequently used is transformation or kernel-based integration, which consists of transforming each omic data into an intermediate form, usually taking the shape of a relationship matrix between the individuals [8–11]. Effects owing to different omics can then be integrated into a single analytical model, each effect being associated to a given kernel. Eventually, kernels can be further combined by the Hadamard product to add extra interaction terms between effects [8, 11]. Integration can also be carried out across models, in what is known as model-based integration [7]. Such integration can happen for a given omic type over different datasets or populations, each one summarized by its own model, with a final global model utilizing the top features contributed by each of the initial models. Another variant of the same model-based integration proceeds through a multistage approach, combining sequentially different omics for a given population [12]. One of the simplest integration approaches, however, remains data concatenation [13], by which multiple omics are placed side by side into a single large input matrix. Unlike kernels, whose results are produced at the individual level, the concatenation allows for the effects of multiple features at each omic to be estimated, whether they are SNPs, transcripts or any other omic. Another advantage, derived from that atomization of effects, is that interactions between omics can be more easily captured, without the risk of being lost by intermediate transformations.

Most of the studies dealing with omics integration for phenotypic prediction have focused on gauging predictive abilities. To that level, the reported benefits are context dependent across studies and, in general, amounting to small differences when compared to single omics counterparts. A series of published comparisons in maize illustrates this point. Using kernels to integrate genomic and transcriptomic data, Guo et al*.* [8] found improved accuracies over single omic approaches for most of the 11 economically important traits under study. In contrast, Schrag et al*.* [9] found no benefit in integration on a similar set of production-related traits. For Azodi et al*.* [13], however, using concatenation of genomic and transcriptomic data for three maize traits yielded benefits for only one trait. Studies on other biological models also showed similar context dependent results. Based on the *Drosophila melanogaster* Reference Panel and different transcriptomic datasets, Li et al*.* [10] and Morgante et al*.* [11] found no advantage of integration following a multiple kernel approach in terms of predictive abilities, and over different sets of fitness-related traits. When the integration included a gene ontology (GO) category as an additional layer of information, accuracies were surprisingly improved [8]. Using the same Drosophila panel, Ye et al*.* [12] also found some benefit in following a model-based integration approach, with an initial modelling stage aiming to detect SNP associated with transcripts (eQTLs), and a subsequent prediction model focused on resulting eQTLs. The number of studies, however, is not yet high enough to draw general conclusions. Benefits may depend jointly on methods of integration and targeted traits, reflecting the complexity of underlying architectures and conditions of studied populations.

Currently, there are still few studies available that focus on the possible causes underlying the benefits brought by omics integration to prediction. Already, at the statistical level, omics such as sequence polymorphisms and transcriptomics are likely non-orthogonal to some extent. Many studies have already illustrated this dependence [14–18], by testing associations between transcript expressions and DNA polymorphisms at given positions (eQTL), and suggesting underlying regulatory mechanisms from the latter. If such dependancy is not conveniently handled at the model level, one can expect inaccurate estimation of effects and impaired prediction accuracy as a result [7, 19]. Redundancy between genomic and transcriptomic data has been addressed in several studies, typically by gauging the amount of extra variance captured by the different integration models compared to single-omic versions. For instance, the successful integration described by Guo et al*.* [8] was systematically accompanied by extra levels of captured variance, suggesting that each additional layer added to the model contributed to some extent to non-redundant information, thus improving the prediction. Conversely, the opposite behaviour is described in Morgante et al*.* [11], with integrative models showing similar levels of captured variance to those of single-omic, indicating high levels of redundancy. It is interesting to note here that for this latter study, redundancy was not found between GO terms, the only layer bringing benefits to integration in the study. The most explanatory GO terms with genomic data were different from those detected for transcriptomic data. Another, more biological, approach is to look to what extent the most important features in both omics show simultaneously mutual associations, in other words, if relevant SNPs are associated or not to relevant transcripts for a given phenotype. Azodi et al*.* [13] showed, in maize, that the transcriptome provides information on the phenotype that is different from that brought by genomic polymorphisms. More precisely, they highlighted that the information carried by the most important transcripts to predict flowering time is not redundant with that of the most important SNPs. In mice, two independent studies used a Bayesian approach to model the phenotype with both genomic and transcriptomic data [20, 21]. These studies showed that specific SNPs (eQTLs) associated with gene expression profiles can contribute to the observed redundancy between the two data sources, which is reflected by the fact that their importance for phenotype prediction was substantially affected under the integrative approach.

Further research is needed in the area of data integration. It is clear that the mechanisms by which integration is successful when predicting phenotypes are still not precisely known over a wide range of conditions and species, with the hypothesis of redundancy being one of the possible explanations. To some extent, redundancy reflects the high level of interconnectivity going from the raw genomic sequence to the organismal phenotype. Both redundancy and interactivity are key to understanding genetic architecture beyond the simple list of effects that is typically provided by genomic approaches. Most studies on data integration involve various species such as drosophila, maize, or humans but here, we propose new insights on data integration for black poplar (*Populus nigra* L.), using one of the simplest integration alternatives (concatenation), combined with one of the most popular prediction approaches (ridge regression). We aimed to evaluate the factors affecting prediction accuracy when integrating genomic and transcriptomic data for phenotype prediction. Using a large number of diverse phenotypes collected from black poplars in two common gardens, we specifically analyzed the change in ranking of effects of potentially redundant predictors (eQTLs and their target genes) between multi- and single-omic model, together with prediction accuracy. For a more functional point of view, we further studied the redundancy using a GO enrichment analysis.

## Results

### Multi-omic model displays performance advantages over the single-omic models for specific functional types of traits

**Fig. 1:**
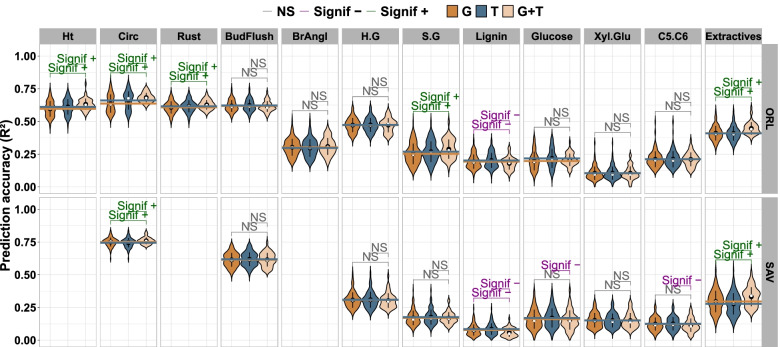
Prediction accuracies. Violin plots of prediction accuracies (R^2^) for 21 traits in the poplar dataset according to three models: genotypic data only (G model coloured in dark brown to the left in the panels), transcriptomic data only (T model coloured in dark blue), and concatenating both genotypic and transcriptomic data (G + T model coloured in light brown to the right). Distribution of accuracies resulted from a cross-validation scheme. Significance from paired tests is shown for comparisons between models, with a sign indicating if the accuracy was increased (+) or decreased (-) in the multi-omic model in comparison with the single-omic. Some traits were evaluated at two sites (“ORL” standing for Orleans in France and “SAV” for Savigliano in Italy). The white and black dots show the median and mean of the precision distributions, respectively. The dark brown and dark blue horizontal lines represent the mean of precision distributions of G and T models, respectively

Twenty-one traits were phenotyped (Table 1) on 241 poplars grown in two common garden experiments located at contrasting sites (Orleans, France, and Savigliano, Italy). RNA sequencing analysis was also performed on young differentiating xylem and cambium tissues of the entire set of genotypes sampled at the Orleans site, resulting in large genomic (428,836 SNPs) and transcriptomic (34,229 transcripts) datasets. For each phenotypic trait, three ridge regression models were built: firstly, with genotypic data as predictors (denoted G), secondly, with transcriptomic data as predictors (T), and thirdly, with integration by concatenation of both omics data (G + T). The prediction accuracies (R^2^) for the three models varied across trait types, with growth, pathogen tolerance and phenology traits having average performances above 0.5 at both testing sites, while biochemical and architectural traits showed average performances below 0.5 (Fig. 1).

**Table 1:** List of phenotypic traits

Functional types	Trait	Abbreviation	Site	Year
Growth	Height	Ht	ORL	2011
	Circumference	Circ	ORL	2011
			SAV	2009
Pathogen Tolerance	Tolerance to rust	Rust	ORL	2009
Phenology	Date of bud flush	BudFlush	ORL	2009
			SAV	2011
Architecture	Branching angle	BrAngl	ORL	2009
Biochemical	H/G lignin ratio	H.G	ORL	2011
			SAV	2009
	S/G lignin ratio	S.G	ORL	2011
			SAV	2009
	Lignin content	Lignin	ORL	2011
			SAV	2009
	Glucose content	Glucose	ORL	2011
			SAV	2009
	Xylose to glucose ratio	Xyl.Glu	ORL	2011
			SAV	2009
	5C/6C carbon sugar ratio	C5.C6	ORL	2011
			SAV	2009
	Extractives content	Extractives	ORL	2011
			SAV	2009

List of phenotypic traits used in the study with their abbreviations, classified by functional type, with site of measurement and year

We compared the prediction accuracies of single- and multi-omic models for each trait, and tested for significant differences with a paired Wilcoxon signed-rank test. For all traits, the differences between the average accuracy of multi-omic model compared to the single-omic ranged from -0.025 to 0.054. Seven of the 21 traits showed a significant gain with the multi-omics model. These 7 traits included all the growth traits, the pathogen resistance trait, as well as 3 of the 14 biochemical traits (S.G_ORL, Extractives_ORL and Extractives_SAV). It is noteworthy that most of these traits (5/7) were measured in Orleans, the site where transcriptomics data were also collected. The only 2 traits for which the multi-omic model was advantageous at Savigliano (Circ and Extractives) also benefited from the multi-omic model at Orleans. Some traits showed a significant loss of accuracy with the multi-omic model, two when the comparison was against the G counterpart (Lignin_ORL, Lignin_SAV), and four with the T model (Lignin_ORL, Lignin_SAV, Glucose_SAV and C5.C6_SAV). Of note, all these traits displaying a decrease in accuracy with the multi-omics model were biochemical traits, had low prediction accuracies and were more often than not measured in Savigliano (3 in Savigliano versus 1 in Orleans).

In summary, the multi-omic model showed performance advantages over the single-omic models in 7 of the 21 traits, more frequently on traits measured in Orleans where transcriptomic data were collected than in Savigliano. The multi-omic model underperformed compared to the single-omic models on 4 occasions, corresponding to 3 traits measured in Savigliano and one in Orleans. For the 10 remaining traits, no differences between models were detected (Fig. 1, Table S1).

### eQTL analysis sheds light into the interplay between the genome and the transcriptome

**Fig. 2:**
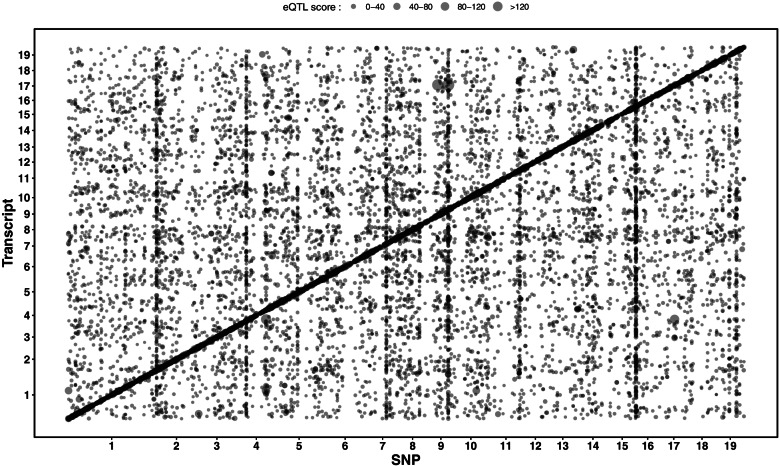
eQTL map between SNPs and transcripts. Map of associations (dots) between SNPs (x axis) and transcripts (y axis) through an eQTLs analysis with a multi-locus model. Dot size reflects the association score (-log_10_ of the p-value of the test) and dot positions correspond to genomic locations of transcripts and SNPs on the 19 chromosomes of the *Populus trichocarpa* reference genome (v3.0). The darkened diagonal includes all *cis* mediated associations, while the off-diagonal dots represent the *trans* associations

To gain further insight into the interplay between the two omic layers for phenotype prediction, we studied their relationships through an eQTL analysis. Such analysis was performed with a multi-locus mixed-model approach which accounted for linkage disequilibrium between SNPs [22]. The resulting eQTLs were further classified into *cis* and *trans* regulatory elements according to their genomic proximity to the transcripts to which they were associated (for more details see the method section). Figure 2 presents a map of these associations with dot size reflecting the eQTL score (-log_10_ of the p-value of the association test). The darkened diagonal includes all *cis* mediated associations, while the off-diagonal dots represent *trans*-eQTLs. It is important to note that some positions at the marker axis present highly populated vertical trails across the genome, corresponding to important regulatory hubs.

We found a total of 18,248 eQTLs for 10,242 out of the 34,229 transcripts available in the transcriptomic dataset. Of these 18,248 eQTLs, 7,845 (43%) were located in *cis*, and 10,403 (57%) were located in *trans* (Fig. S1A). *Cis*-eQTLs displayed on average a larger effect than *trans*-eQTLs (Fig. S1B). The maximum distance between a *cis*-eQTL and its associated gene was 12 kb.

### The ranking of predictor effects was more affected for traits displaying a predictive advantage with integration

To gain insight into the factors explaining the gain or loss in predictive ability when using two omics rather than a single omic, we looked more closely at the variation in ranking of effects of the individual predictors over the two types of predictive models. For each of the three models, the rank was established based on squared effects from the ridge regression models (denoted rank hereafter).

We looked at correlations between the rank of predictors across single- and multi-omic models, splitting the predictors into categories determined by the eQTL analysis: *trans*-eQTLs (10,403 associations), *cis*-eQTLS (7,845 associations), not eQTL (419,414 SNPs), *trans*- regulated transcripts (6,305 transcripts with at least an eQTL in *trans*), *cis*-regulated transcripts (3,573 transcripts with at least an eQTL in *cis*) and no eQTL transcripts (22,796 transcripts for which no eQTL was detected). We looked particularly at how changes in rank occurred across these categories of predictors (Fig. S2). For SNPs, the correlation between the ranks ranged from 0.58 to 0.99 across traits and predictor categories (cis- or trans- eQTLs and regulated transcripts, Fig. S2B). They were generally lower for the traits that also showed advantages with the G + T model over single-omic models, and for those measured in Orleans. Rust resistance, for instance, had the lowest correlations across the different categories among all measured traits (0.58, 0.62 and 0.64, respectively for *trans*-eQTLs, *cis*-eQTLs and non-eQTLS). Also, growth traits showed relatively low correlations compared to other traits, although this happened only for measurements in Orleans (Ht_ORL, Circ_ORL), with those in Savigliano (Circ_SAV) being much higher and comparable to the top correlations. For the remaining traits, correlations between ranks remained high, generally above 0.9 but with a few values close to 0.8 (Fig. S2B). The correlations between transcript ranks were generally lower than those for SNPs, varying between 0.51 and 0.9 across traits and predictor categories (Fig. S2D). Following a similar pattern as for SNPs, the traits showing the lowest correlations were also those for which the multi-omic models displayed a predictive advantage over single omic models, as well as those measured in Orleans. Growth and rust resistance showed the lowest correlations. Although with small differences, *cis*-regulated transcripts showed lower correlations than those from *trans*-regulated counterparts, across traits and sites.

### *Trans*-eQTLs show the most important changes of squared effect rank between multi- and single-omic models

Previous correlations pointed to changes in rank in some of the categories of predictors. Thus, we decided to compute differences in rank between the multi-omic model and that of the single-omic model (either T or G, for transcripts and SNPs, respectively) in order to gain insights into how this could affect trait predictability (see Methods for details).

When looking at the variation of the differences in rank (Fig. S3, Fig. S4), the amounts were much larger for eQTLs (G + T versus G) than for targeted transcripts (G + T versus T). Higher variations were also found for *trans*-eQTLs than for *cis* counterparts, and for traits phenotyped at Orleans compared to those phenotyped in Savigliano. Thus, changes in rank occurred with more intensity for eQTLs with a TRANS regulation, and when linked to traits measured where the transcripts were sampled.

**Fig. 3:**
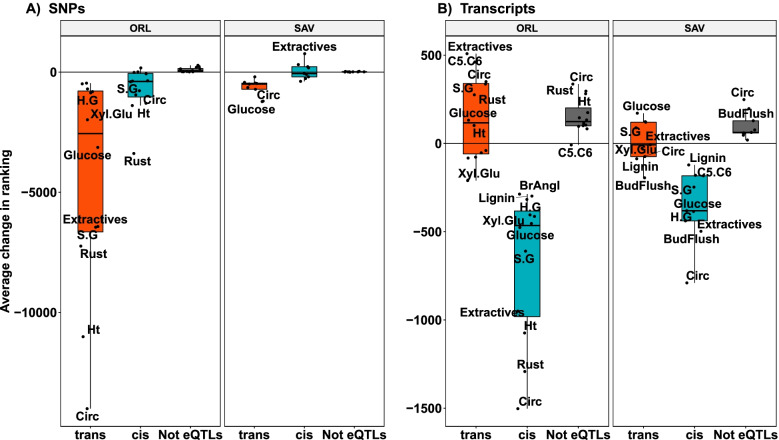
Distribution of change in predictor rank. Boxplot of the average change in rank of SNPs (panels **A**) and transcripts (panels **B**). Each dot represents the average difference per trait, per site of the predictor ranks between the multi-omic model (G + T) and the single-omic models (G for SNPs and T for transcripts). The red and blue boxplots show the distribution of the average rank change for the *trans*-eQTLs and *cis*-eQTLs (**A**) or *trans* regulated transcripts and *cis* regulated transcripts (**B**), respectively. The boxplot in black shows the distribution for the predictors that have not been found to be associated in the eQTL analysis

An alternative way of visualizing these changes is represented in Fig. 3. Here, changes were averaged for a given trait and the resulting distribution of averages represented by predictor category and site. Patterns were very different between eQTLs and targeted transcripts, and also between sites. The most important changes in ranking happened at the Orleans site. With respect to predictor typologies, it was *trans*-eQTLs that showed the most significant changes, with an overall decrease in rank when switching to the G + T model, notably for the traits benefiting the most in performance from concatenation (growth and rust resistance). Less conspicuous were the changes for *cis*-eQTLs, which were negative overall but of lesser magnitude. Non-eQTLs showed generally small changes across traits. For targeted transcripts, the most impacted typology was CIS regulated genes, with an overall loss in ranking across traits.

### A negative relationship exists between the change in ranking of *trans*-eQTLs and *cis*-regulated transcripts and the predictive ability of the integrated multi-omic model

**Fig. 4:**
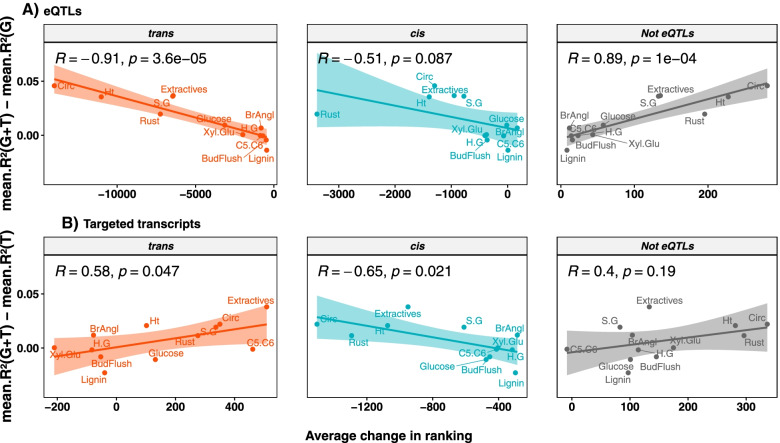
Relationship between change in predictor rank and muti-omic prediction advantage. Regression across traits measured at Orleans between average change in rank of predictors and advantage in performance of the multi-omic model (G + T) over the single-omic counterpart (G for SNPs and T for transcripts). The top panel (**A**) shows the regression obtained with the eQTLs (*trans*-eQTLs on the left, cis-eQTLs in the middle, and SNPs not detected as eQTL on the right). The bottom panel (**B**) shows the regression obtained with the regulated transcripts (*trans* on the left, cis in the middle, and not found to be associated with eQTLs on the right)

Figure 4 represents the link across traits evaluated in Orleans between average change in rank of predictors and advantage in performance of the multi-omic model over its single-omic counterpart. In the case of *trans*-eQTLs, generally the most affected predictors following concatenation, a significant relationship (*r* = -0.91, *p* = 3.6e-05) was able to be drawn where gains in prediction occurred at the expense of decreases in ranking of predictors. The opposite was found for SNPs that did not display any association with transcripts (non-eQTLs) with a positive and significant correlation (*r* = 0.89, *p* = 1e-04). When we categorized all SNPs within a 500 bp around the top SNPs in the same way as the top SNPs themselves, these correlations remained significant and a significant negative correlation was also found for *cis*-eQTLs. However, this correlation for *cis*-eQTLs was found only for a 500 bp window and not for larger ones, while the significant correlations for *trans*-eQTLs and not eQTLs were maintained with windows as large as 5 and 25 kb, respectively (Fig. S5). A similar pattern to *trans*-eQTLs, although of lesser magnitude, was found for *cis-*regulated transcripts (*r* = -0.65, *p* = 0.021). On the opposite, a significant positive link was found when considering *trans-*regulated transcripts at Orleans, while at Savigliano no significant link could be detected across all categories of predictors (Fig. S6).

### Gene ontology analysis suggests that top targeted transcripts or eQTLs are trait specific

We further selected the transcripts or eQTLs whose ranks were most affected by data integration, by focusing on the 2.5% percentile at each extreme of the distributions, and carried out an enrichment analysis in GO terms on the selected features. The complete GO analyses for all traits, with complete terms and IDs are available in Table S2.

**Fig. 5:**
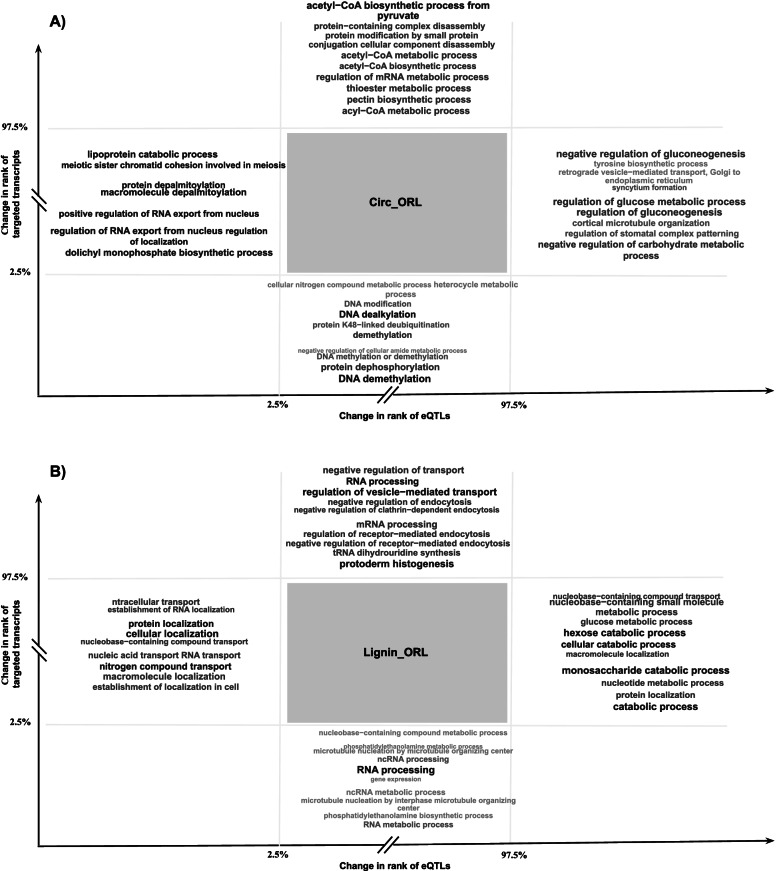
Gene ontology (GO) terms enrichment analysis. Schematic representation of the enriched GO terms among the top targeted transcripts or eQTL gene models list for **A**) the circumference of the tree trunk or **B**) the lignin content, both evaluated at Orleans. Font size and grey intensity are proportional to -log_10_(p) of the top 10 GO terms

The analyses of enrichment terms offered the following outcomes, depending on traits and features involved. For all the analysed traits, both those gaining from the multi-omic approach and those without gains or losses, we detected enrichment in GO terms from general processes for those eQTLs (e.g. “regulation of RNA export”, “regulation of nucleobase”, “positive regulation of RNA export”, lipoprotein catabolic process”) and targeted transcripts (e.g. "DNA dealkylation" or "DNA demethylation" for Circ_ORL, or "cellular localization" and "RNA processing" for Lignin_ORL) having the most negative impacts on their ranking during integration (leftmost and lower lists in Fig. 5A and Fig. 5B, below 2.5% on horizontal and vertical axes).

For traits gaining from the multi-omic approach, however, we identified enrichment in GO terms that were specific to the trait and for those features, eQTLs and targeted genes, that showed the most positive impacts on their ranking during integration (rightmost and upper lists in Fig. 5A, above 97.5% on horizontal and vertical axes). Some examples of such specific GO terms identified for Circ_Orl were:, "pectin biosynthetic process" or "acetyl-CoA biosynthetic process" for targeted genes, and “regulation of glucose metabolic process” or “negative regulation of cellular carbohydrate metabolic process” for eQTL (Fig. 5A).

Finally, for the traits not showing a benefit from the multi-omic approach we captured enrichment in GO terms from general processes for those eQTLs and targeted transcripts having the most positive impacts on their ranking during integration (rightmost and upper lists in Fig. 5B, above 97.5% on horizontal and vertical axes). Examples of such enrichments for Lignin_ORL were: "RNA processing" or "regulation of vesicle-mediated transport" for targeted genes, and "catabolic process " or "monosaccharide catabolic process" for eQTL..

In summary, only the features with positive impacts on their ranking for traits benefiting from multi-omic showed a different behaviour from the other scenarios, with enrichment in GO terms that seemed to be specific to those traits, and therefore potentially useful for prediction.

## Discussion

In this study, we used 21 traits to compare the relative advantages of integrating genomic and transcriptomic data for phenotype prediction versus using each omic separately. This relative advantage of integration over a single-omic approach varied across traits. For such traits as growth and pathogen resistance, integration yielded more accurate predictions than the single-omic counterparts. For most of the other traits, principally biochemical traits, concatenation gave no advantage or even, in few cases, poorer performance than using a single omic. By using a simple modelling approach like ridge regression, we showed that the gains in the traits that benefited from integration were associated with systematic changes in rank for some specific predictors, and that those predictors were involved in the interplay between SNP polymorphisms and transcripts, thus pinpointing adjustments in effects due possibly to redundancies. Such findings at the statistical level were also strengthened by a subsequent biological analysis of GO terms.

In order to better understand the reasons underlying trait differences in the benefits of concatenation, we sought to evaluate the interplay between the genomic and transcriptomic data, by making use of an eQTL analysis. Such analysis allowed us firstly to categorize the predictors into *cis*-eQTLs, *trans*-eQTLs, not-eQTLs, *cis* regulated transcripts, *trans* regulated transcripts and transcripts with no eQTL detected. Secondly, based on this categorization, we could quantify the changes in predictor rank within each of these categories when using the multi-omic model through comparison with the single-omic models.

Across all the traits under study, we found a strong, negative and significant correlation between the relative advantage of the multi-omic model compared to the single-omic and the decrease in rank (importance) of the predictors mostly for *trans*-eQTLs (*r* = -0.91) and to a lesser extent for *cis*-eQTLs (*r* = -0.6 when using a 500 bp window around the top SNP) and *cis-*regulated transcripts (*r* = -0.65). Such a relationship could be interpreted in terms of redundancy between predictors coming from different omics. Indeed, the traits that benefited the most from concatenation were also those for which strong falls in ranking for *trans*-eQTLs were observed in the combined predictive model compared to the single-omic counterparts. Therefore, those predictors already involved in associations between SNPs and transcripts (eQTLs and their target genes) were also the ones mostly affected at the integrative model, when their covariation or redundancy with other features could have undermined their contribution to prediction. Redundancy, per se, would not necessarily explain gains or loss in performance, but down weighting redundant predictors could allow other minor predictors, otherwise silenced, to increase their rank in such a way that the concatenation model improves in predictability. This point is underlined by the positive correlation detected between change in rank of SNPs not found to be associated with transcripts and a trait prediction advantage with multi- over single-omic models.

To our knowledge, this study is the first to establish such a relationship between integration success and change in rank of effects from interconnected omic layers, pointing eQTLs as key players in such interplay. It is worth mentioning that we could establish such a relationship because of the relatively large number of traits under study, compared to previous works [8–11, 13, 20, 21]. The relative gains from integration, estimated in R^2^ between observed and predicted values, ranged from -0.02 to 0.05 across all 21 traits. These gains are indeed small, but consistent with the state of the art. Despite the small advantages, differences between traits were sufficiently important to reveal discriminant patterns in the ranking of important predictors before and after integration. Our objective here was to better understand the factors that underlie this change in ranking and produce new knowledge that will allow us to further improve the benefits of integration in more consequential ways with other methods.

### A gain of integration was mainly found for traits related to growth and for traits evaluated in the same location as transcriptomics

We observed a significant advantage of multi-omic over single-omic models for all growth-related traits (Ht_ORL, Circ_ORL and Circ_SAV). Since growth results from cell division and expansion in the apical and cambial meristems (xylem and cambium) [23] this relationship between the tissues from which we extracted transcripts and the growth traits (circumference and height) may explain the significant integration advantage observed for these traits.

The advantage of integration over the single-omic models was also observed for leaf rust resistance. Although xylem and cambium, the tissues sampled for RNA sequencing, seem disconnected to a phenomenon occurring at the leaf level, the relationship here is likely indirect since links between resistance and growth have been reported by other studies [24–26]. Following the same reasoning, phenology and architectural traits considered here do not show clear relationships with cambial meristems or with growth-related traits, and therefore support the lack of benefits observed for them in the concatenation models.

For the majority of wood biochemical traits, the multi-omic integration model performed similarly or worse than the single-omic model. It is noteworthy that these traits are overall not well predicted with either model. One of the reasons for that poor performance could be that these traits are in fact near infrared spectroscopy predictions, which may include some extra noise that affects their prediction.

These series of observations across traits also point to the idea that transcripts capture some new information not necessarily available for SNPs. These may include non-additive (gene–gene) or genotype-environment interactions occurring in the specific tissue sampled for transcriptomics, both of which are not explicitly modelled when using exclusively SNPs as predictors.

Among the 21 analysed traits, we observed that the benefits of the concatenation model were more common for traits measured in the same location as transcriptomic data collection (Orleans). This advantage can be interpreted in terms of genotype-environment interactions being effectively captured by the transcripts [27, 28]. Conversely, for phenotypes evaluated at Savigliano, the transcriptomic data was more likely to provide information already provided by that of the SNPs, thus negating any advantage of multi-omic integration.

### Negative change in rank between the two models implies a decrease in importance

Our goal was to identify factors that influence the success of genomic and transcriptomic data integration for phenotype prediction. To this end, we chose a simple integration method that allowed us to track rank changes of each variable between the integration and the single-omic models, with decreasing ranks implying losses in importance. As described in Ritchie et al*.* [7] and Zampieri et al*.* [29], there are several ways to integrate multi-omic data, the simplest being the concatenation method. Using a ridge regression model, concatenation allows direct estimation of each predictor effect, accounting for all other variables (SNPs and transcripts), unlike LASSO and elastic-net where some degree of variable selection is applied, while trying to minimize the covariation between the predictors' effects. This method allowed us to track the evolution of predictor ranks in the multi-omic compared to the single-omic models, thereby inferring potential redundancies by the changes in rank. Since the number of SNPs was about 12.5 times higher than the number of transcripts, the effects of SNPs or transcripts between the two models were not at the same scale. We thus needed a scale-free system for making comparisons. A simple and efficient way to do this was to work with ranks of the squared effects of predictors.

Comparing the changes in rank between the multi- and single-omic models was indicative of the gain or loss of importance of each predictor. A predictor will have a positive change in rank when it has low importance (low rank) under the single-omic models and ends up with high importance (high rank) in the multi-omic model. Conversely, a negative change in rank between the two models implies a decrease in importance. No rank change corresponds to a predictor that keeps the same importance between the two models. Using the average change in rank between models would not work if we were considering all predicting features together as this would remain constant over traits. However, we were focusing on distinct subsets of predictors determined by the eQTL analysis, and this yielded some variation across traits, which seems useful to better understand their predictability.

### Integration success is driven by the fall in rank of predictors involved in covariation between genomics and transcriptomics

Our main hypothesis was that reducing the sources of redundancy between SNPs and transcripts plays an important role in the success of integration. The eQTLs are an ideal source of redundancy between SNPs and transcripts, so we performed an eQTL analysis to identify these within our dataset. It is important to recall that the SNPs were derived from RNAseq, thus capturing SNPs from mainly the intragenic spaces. While, this might have affected our ability to detect some *trans*-eQTLs, our multi-locus analysis showed that *trans*-eQTLs remained the majority, with some hotspots or hub loci associated with a fairly large number of transcripts. Such overabundance of trans—eQTLs has previously been reported in other species such as yeast [17], maize [30, 31], or tomato [16], although cases with similar or fewer occurrences also exist [14, 18].

The main results of our study are the strong, negative and significant correlation found between the relative advantage of the multi-omic model over the single-omic and the average fall in ranking (loss in importance) of the trans-eQTLs and to a lesser extent the cis-regulated transcripts. To our knowledge, this study is the first to establish this direct relationship between integration success and changes in ranking of eQTLs and regulated genes. This relationship was possible to determine due to the relatively large number of diverse traits that we used.

Previous studies have suggested a key role of eQTLs for multi-omic integration between genomics and transcriptomics. Ehsani et al*.* [20] observed in mice losses in importance of eQTLs in the combined genomics and transcriptomics model versus the model with only genomics for a phenotype showing an advantage of integration (body mass). Such behaviour of eQTLs in this study was observed only for a single phenotype with low resolution genotyping data. Also, Ye et al*.* [12] were successful in improving the performance of phenotype prediction in Drosophila using genotypes of eQTLs regulating genes important for the phenotype. They proceeded with successive selection steps involving a transcriptome wide association study (TWAS) with an eQTLs analysis for the TWAS significant genes, while optimizing the detection thresholds of these two analyses. Their results suggest a key role of eQTLs for the integration between genomics and transcriptomics. Azodi et al*.* [13] observed in maize that concatenation between genomic and transcriptomic data improved the prediction of one out of 3 studied phenotypes. For this phenotype, they showed the most important SNPs and transcripts were not redundant in the sense that they were not located in the same genomic regions, nor were they regulators of important transcripts.

### The observed redundancy may be explained by biological processes

Until now, we have shown statistically that certain predictors involved in covariation between omics were penalized with a fall in rank under integration. Our GO enrichment analysis seemed to provide a more biological point of view with further evidence of the role of redundancy. Overall, genes representing general ubiquitous biological processes were more likely to be a source of redundancy, while those associated with specific processes provided more useful information to the prediction process. The GO analysis showed that eQTLs and targeted genes lowering their rank under integration were by and large associated with specialized processes relevant to the predicted phenotype. This pattern was observed particularly for traits which also benefited the most from integration. In contrast, the eQTLs and targeted genes whose rank was most heavily affected under integration showed a characteristic enrichment of terms linked to general ubiquitous biological processes, such as cell cycle. As the transcriptomic data came from young differentiating xylem and cambium tissues, the redundancy (and complementarity) we observed was strongly associated with phenotypes related to wood production, e.g. trunk circumference. This interpretation may also apply to the loss of prediction accuracy for traits whose genes were unlikely to be represented in our transcriptome, such as rust resistance. One eventual validation could be to complement the transcriptomic data with a tissue less connected to xylem and cambium, e.g. leaves, and focus on traits more specifically expressed in that tissue, such as rust resistance. If the genes associated with general biological processes are found to be sources of redundancy through GO analysis, one strategy to improve prediction could be to reduce or minimize their contribution to the models.

### Perspectives

For the sake of simplicity, our study could not take the extra step to devise a novel alternative to account for such redundancies in the prediction model. However, here we outline a basic strategy whereby the contribution of predictors to the prediction model is penalized based on how redundant they are within the remaining data. Under kernel-based integration, for instance, some kind of optimization of composition in features included in the relatedness matrices could be devised so that the resulting kernels bring complementary information. Under a model-based integration, a multistage approach could be devised where associations between all involved omics are firstly carried out, so that the features contributing the most to the associations can be subsequently penalized to some degree or filtered out when it comes to construct a consensual model. More research is required to devise and test a strategy to derive robust weightings.

It would be essential to gather further information on the beneficial role of multiple omics, collected at different development stages or distinct tissues, to enable linkingto different traits. Despite being a costly endeavour, such integration studies on specific training populations would allow us to identify important hubs in the genetic architecture of traits, and enable differential weighting on other, related populations with no or basic access to additional omic layers.

## Conclusions

One of the main findings of this study was the fact that certain predictors with ubiquitous connections seem to be made redundant when integration took place. An additional gene ontology (GO) enrichment analysis appeared to corroborate this statistical output. These two complementary approaches showed empirically over a series of traits how the best predicting scenarios are built, excluding certain features while promoting others according to their redundancies within the data. To our knowledge, this is a novel finding delineating a promising method to explore data integration.

## Methods

### Plant material, experimental design and phenotypic evaluation

We studied 241 genotypes of *Populus nigra* originatingd from 11 major river catchments across 4 countries and representative of the species range in Western Europe. More details on the origin of these genotypes including their depositary are available in the GnpIS Information System [32] via the FAIDARE data portal (https://urgi.versailles.inra.fr/faidare/), using the keys "Black poplar" and "POPULUS NIGRA RNASEQ PANEL" for the fields "Crops" and "Germplasm list", respectively. These poplars were evaluated in common garden experiments located on 2 contrasting sites (Orleans, denoted ORL and Savigliano, denoted SAV), as underlined by large differences in growth [33, 34]. At each site, the experimental design consisted of a randomized complete block design with 6 blocks, and thus 6 repetitions per genotype. Twelve traits were evaluated on the 2 sites, as previously described [34, 35]. We considered traits measured at the 2 sites as different traits, leading to a total of 21 traits (detailed in Table 1). These traits can be categorized into 5 types: growth, pathogen tolerance, phenology, architecture, and biochemistry. At Orleans, the trees were grown through 3 successive cycles: 2008–2009, 2010–2011 and 2012–2015. During the first growth cycle (2009), rust tolerance (Rust) was assessed with a discrete score from 1 (no symptoms) to 8 (generalized symptoms), as detailed in [36]. Average branch angle (BrAngl) was evaluated with a score on proleptic shoots from 1 to 4 (score 1: between 0° and 30°; score 2: between 30° and 40°; score 3: between 40° and 55°; score 4: and between 55°and 90°). During the second growth cycle, height (Ht) and circumference at 1-m above the ground (Circ) were measured on 2 year-old trees (winter 2011). At Savigliano, trees went through two cycles: 2008–2009 and 2009–2010. Only Circ was measured during the second growth cycle on 2 year-old trees (winter 2010). Biochemical traits consisted of predictions of several chemical compounds obtained from near-infrared spectra on wood samples collected in the same years as growth traits and at both sites, as described in Guet et al*.* [33]. Biochemical traits included: extractives content (Extractives), total lignin content (Lignin), ratios between different lignin components like p-hydroxyphenyl (H), guaiacyl (G) and syringyl (S) (H.G, S.G), total Glucose content (Glucose), ratio between xylose and glucose content (XylGlu) and the ratio between 5 and 6 carbon sugars (C5.C6). One phenological trait was also measured, BudFlush as discrete scores for a given day of the year, measured on the apical bud [37].

### Phenotype adjustments

All 21 traits were independently adjusted to field micro-environmental heterogeneity using the breedR package [38]. The model included blocks and spatial effects (autoregressive residuals function) to account for micro-environmental heterogeneity. A model selection was also carried out using the AIC to select the effects to be included in the model and to adjust the autoregressive parameters. The genotypic adjusted means from these models were used as the phenotypes for this study.

### Genotype and transcriptomic data

RNA sequencing was carried out in 2015 on young differentiating xylem and cambium tissues collected from two replicates of the 241 genotypes located in two blocks of the Orleans design [. These two tissues corresponded to the location in the tree where wood production and differentiation occur. We obtained sequencing reads for 459 samples corresponding to 218 genotypes with two replicates and 23 genotypes with 1 replicate. These sequencing reads were used to provide both transcriptomic and genomic data.

For transcriptomic data, the reads were mapped on the *Populus trichocarpa* v3.0 primary transcripts using bowtie2 v2.4.1 [39] and read counts were retrieved for 41,335 transcripts with home made scripts. Only transcripts with at least 1 count in 10% of the individuals were kept, yielding 34,229 features. The raw count data were normalized by Trimmed Mean of M-values using the R package edgeR v3.26.4 [40] and we calculated the counts per millions [41]. To make the CPM data fit a Gaussian distribution, we computed a *log*_2_(*n* + 1) instead of a *log*_2_(n + 0.5) typically used in a voom analysis [41], to avoid negative values, which are problematic for the rest of the analysis. For each transcript the log_2_(n + 1) of the CPM were fitted with a mixed model including experimental (batch) and genetic effects to extract their genotypic BLUPs (Best Linear Unbiased Predictors). These genotypic BLUPs of transcripts were used for the rest of our analysis.

The full details of SNP discovery and genotyping are given in Rogier et al*.* [42], including software used for the different steps, data filtering criteria and final SNP selection (see Fig. 1 in Rogier et al*.* [42] for a schematic representation of the pipeline). Briefly, genotyping data was obtained, first by mapping the RNAseq reads on the *P. trichocarpa* reference genome (v3.0) [43] using BWA-MEM v0.7.12 [44]. After mapping, the SNPs were called using 4 callers. In order to generate a high-confidence SNP set we selected only the SNPs identified by at least 3 of the 4 callers and with less than 50% of missing values. Remaining missing values were imputed using complementary genotyping data obtained with a 12 k Illumina Infinium Bead-Chip array [45]. We then detected 874,923 SNPs. From these, 428,836 SNPs were retained for this study after filtering for a minimum allele frequency of 0.05.

### eQTLs analysis

eQTLs analysis was performed using the Multi-Loci Mixed-Model (MLMM) approach] and implemented in the R package MLMM v0.1.1. MLMM uses a step-by-step forward inclusion and backward elimination approach under a mixed-model framework which accounts for the confounding usually attributed to population structure with a random polygenic effect. For each of the 34,229 transcripts we ran MLMM for up to 10 steps and identified the optimal model according to the mBonf criterion (all selected SNPs are significant at a 5% Bonferroni corrected threshold).Based on the positional proximity of the genes, the significant SNPs detected with MLMM were classified as *cis* regulatory elements (DNA variation regulating the transcription of neighboring genes), and/or as *trans* regulatory elements (regulating the transcription of distant genes), according to the following rules:

- all SNPs associated with the expression of a gene located in a different chromosome are classified as *trans*, and the targeted gene is classified as a *trans* regulated gene;
- all SNPs located on the same locus as the gene they target, according to the genome annotation, are classified as *cis*, and the targeted gene is also classified as *cis* regulated gene;
- the remaining SNPs whose target gene is on the same chromosome but not on the same locus, were split into *cis* or *trans* according to their distance to the middle of the gene they target. We estimated the maximum distance between the *cis*-eQTLs identified in the previous step and the middle of the gene they target as 12 kb (eQTL being on the same position as its target gene). If the distance between SNPs and the gene they target is greater than 12 kb they were classified as *trans*-eQTLs and target gene *trans*. Otherwise, the SNPs and the target gene were classified as *cis*.

### Models, prediction accuracy and cross-validation

Two ridge regression models were built for each trait with a single omic as predictor: genotypic data (**G** model), or transcriptomic data (**T** model), respectively with *p* = 428,836 SNPs and q = 34,229 transcripts’ expression levels variables. A third multi-omic was also built with integration by concatenation of both omics data (**G + T** model). These 3 models can be written as:

1 $$Y={\varvec{X}}\beta +\epsilon$$

where for models **G**,**T** and **G + T**, **X** represent the genotyping matrix (*n* × *p*), the transcript expression level matrix for the genes (*n* × *q*) and the concatenated transcript expression level and genotyping matrix (*n* × (*p* + *q*)). With the same logic, β represents the vector of effect sizes of variables of those matrices. Y is the vector of phenotype, and *ϵ* the vector of residual errors of the model.

The models were computed using the R package glmnet [46] in a 10 inner-fold and 5 outer-fold nested cross-validation framework [47]. The sampling process for the different folds was repeated 50 times. Each cross-validation sample was used across all traits, for all three models. Paired t-tests in R (rstatix package version 0.7.0) [48] were used for comparisons of model performance.

The model performances were measured using *R*^2^ between observed and predicted values.

### SNPs and transcript effects ranking

In order to study the changes operated for each feature (SNP or gene) when changing prediction models from single-omic to the concatenated counterpart, we compared the change in ranking of the effects across models. Ranks were obtained for each predicting model and trait from the ordering of squared effect sizes.

For each variant category, the estimated effect rank was compared between the single-omic model (**G** or **T**) and the multi-omic model (**G + T**) with a paired Wilcoxon test and a Pearson correlation.

The difference in effect ranking between the models was calculated for the different sets of predictors (*cis-* or *trans-*eQTLs or regulated transcripts, and not eQTLs). We also considered windows of increasing sizes around significant SNPs to evaluate the robustness of our findings when extending the *cis*- and *trans*-eQTL categories to neighbouring SNPs. This ranking difference was then averaged for each trait and regressed with the concatenation advantage of each trait, which is the average accuracy difference between the concatenation model and single-omic models:

2 $${\Delta }_{predictor}=\frac{1}{\omega }\sum_{i=1}^{\omega }\left(\begin{array}{c}{R}_{i}\\ \left(G+T\right)\end{array}-\begin{array}{c}{R}_{i}\\ (G or T)\end{array}\right)$$

where predictor represents either SNPs or transcripts, R is the ranking vector of squared effect sizes of the given predictor, *ω* is the number of predictors (p for SNPs and q for transcripts), ${\Delta }_{predictor}$ is the average difference in effect ranking between the multi-omic model and single-omic model by trait for the given set of predictors.

### GO analysis

Functional enrichment was conducted based on the gene ontology (GO) terms associated with the best *Arabidopsis thaliana* homolog and based on the Phytozome v12.1.6 database (32). GO analysis was conducted using R package topGO 2.44.0 [49] and Fisher's exact test with ‘elim’ used to correct for multiple comparisons using only the xylem/cambium expressed genes. The p-value significance threshold for GO terms was *0.05*.

## Supplementary Information

**Additional file 1: FigureS1. **Proportion and score of *cis*- and *trans-*eQTLs. Proportions(A) and scores (B) of *cis-* and *trans*-eQTLs detectedwith the multi-locus approach. Scores correspond to the -log_10_ ofthe *p*-value of the test.

**Additional file 2: Figure S2.** Comparison between the rank of predictors across single- and multi-omic models. Panels A and C represent the boxplots of SNPs and transcripts rank, according to the traits and sites. Features are grouped into the following categories determined after the eQTL analysis: cis or trans eQTL or regulated transcripts and not eQTLs. Panels B (SNPs) and C (transcripts) represent the correlations between the ranks of predictors across single- and multi-omic models, splitting the predictors into the same previous categories, determined from the eQTL analysis. Ranks were computed from squared effects of features in the ridge-regression models.

**Additional file 3: Figure S3.** Variation of the change in rank of the eQTLs and targeted transcripts. Barplot of the variance of change in rank of eQTLs (top) and targeted transcripts (bottom) for each trait (panel) and site (dark grey: Orleans ; light grey: Savigliano). Ranks were computed from squared effects of features in the ridge-regression models.

**Additional file 4: Figure S4.** Change in rank of the eQTLs and their corresponding targeted transcripts. Scatter plots of the changes in rank of the eQTLs and their corresponding targeted transcripts for each trait. Ranks were computed from squared effects of features in the ridge-regression models. The red and blue dots represent the regulation in *trans*and *cis*, respectively.

**Additional file 5: Figure S5. **Stability of the relationships between change in predictorrank and muti-omic prediction advantage for traits measured at Orleans. The relationship is measured as the correlation coefficient between change in rank and relative advantage in prediction with the multi-omic model over the single-omic counterpart. The stability of the relationship is evaluated with respect to the categorization of eQTLs into *cis*, *trans*, or not eQTLs, being defined according to windows of increasing size in bp around the SNPs detected with the multi-locus model.

**Additional file 6: Figure S6.** Relationship between change in predictor rankand muti-omic prediction advantage for traits measured at Savigliano. Regression across traitsmeasured at Savigliano between average change in predictor rank and advantage in performance of the multi-omic model (G+T) over the single-omic counterpart. Ranks were computed from squared effects of features in the ridge-regression models. The top panel (A) shows the regression obtained with the eQTLs (*trans*-eQTLs on the left, *cis*-eQTLs in the middle, and SNPs not detected as eQTL on the right). The bottom panel (B) shows the regression obtained with the regulated transcripts (*trans* on the left, cis in the middle, and not found to be associated with eQTLs on the right).

**Additional file 7: Table S1.** Prediction accuracies compared between multi-and single-omic models.

**Additional file 8: Table S2. **Complete gene ontology analysis of all traits.

## Data Availability

The datasets supporting the conclusions of this article have been deposited in the INRAE dataverse repository (https://doi.org/10.15454/8DQXK5). Information about the RNA-seq project, from which the gene expression data came, is available in the Gene Expression Omnibus (GEO) from NCBI (accession number: GSE128482). Raw sequences (FASTQ) are available in the Sequence Read Archive (SRA) from NCBI (accession number: SRP188754). Information on the studied genotypes is available in the GnpIS Information System [32] via the FAIDARE data portal (https://urgi.versailles.inra.fr/faidare/), using the keys "Black poplar" and "POPULUS NIGRA RNASEQ PANEL" for the fields "Crops" and "Germplasm list", respectively. The code for running the analyses is available at: https://github.com/Tawfekh/Code-Article-Multi-omics-prediction

## Abbreviations

AIC: Akaike Information Criterion
*cis*: DNA variation regulating the transcription of neighboring genes
CPM: Count Per Million
DNA: Deoxyribonucleic Acid
eQTL: Expression Quantitative Trait Loci
G: SNP based ridge regression model
G + T: SNP and Transcript based ridge regression model
GO: Gene Ontology
LASSO: Least Absolute Shrinkage and Selection Operator
MLMM: Multi-locus mixed-model
ORL: Orleans
RNA: Ribonucleic acid
RNAseq: Ribonucleic acid sequencing
SAV: Savigliano
SNPs: Single Nucleotide Polymorphism
Step_0: Initial association scan of MLMM
Step_opt: Optimal association model from MLMM
T: Transcript based ridge regression model
*trans*: DNA variation regulating the transcription of non-neighboring genes
TWAS: Transcriptome wide association
