# Supplementary figures and images for: eQTLs are key players in the integration of genomic and transcriptomic data for phenotype prediction
Source: BMC Genomics. 2022 Jun 28;23:476. doi: 10.1186/s12864-022-08690-7 (PMC9238188; doi:10.1186/s12864-022-08690-7)

A)

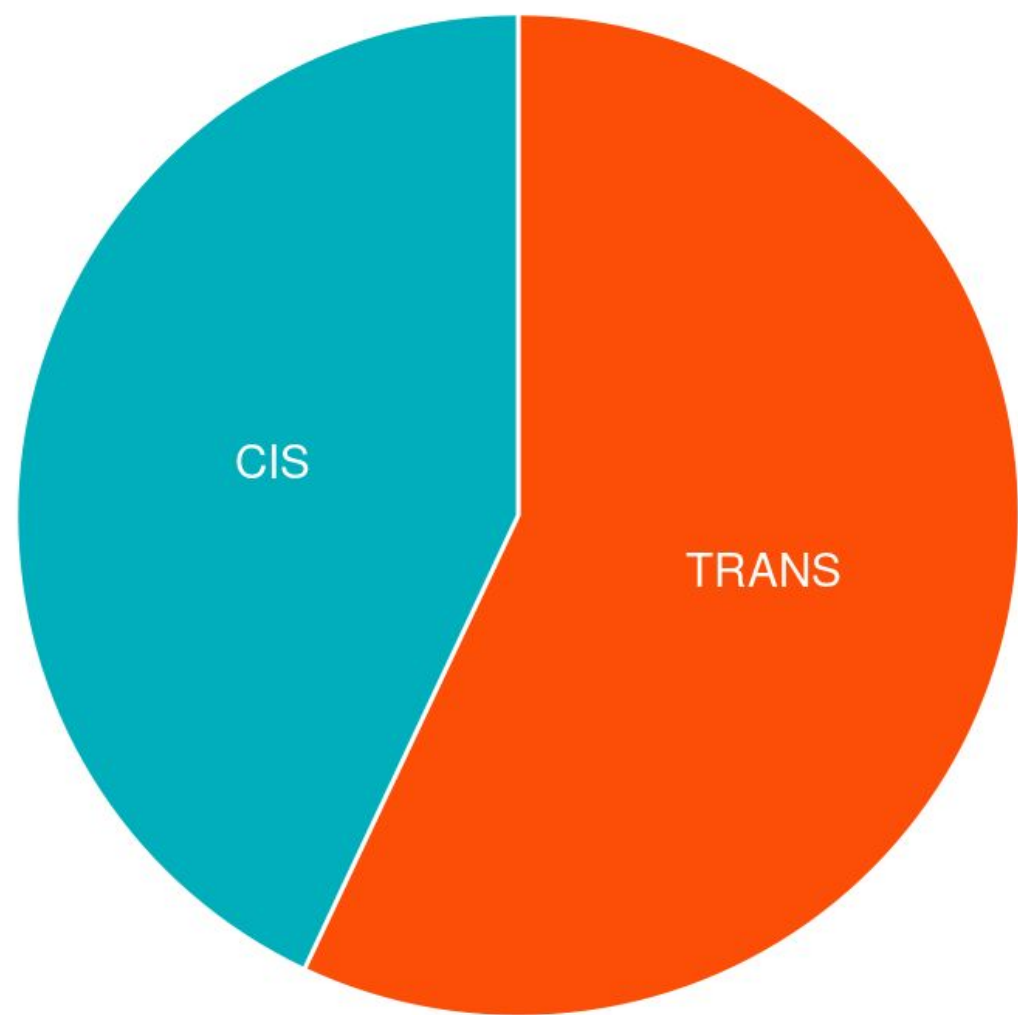

B)

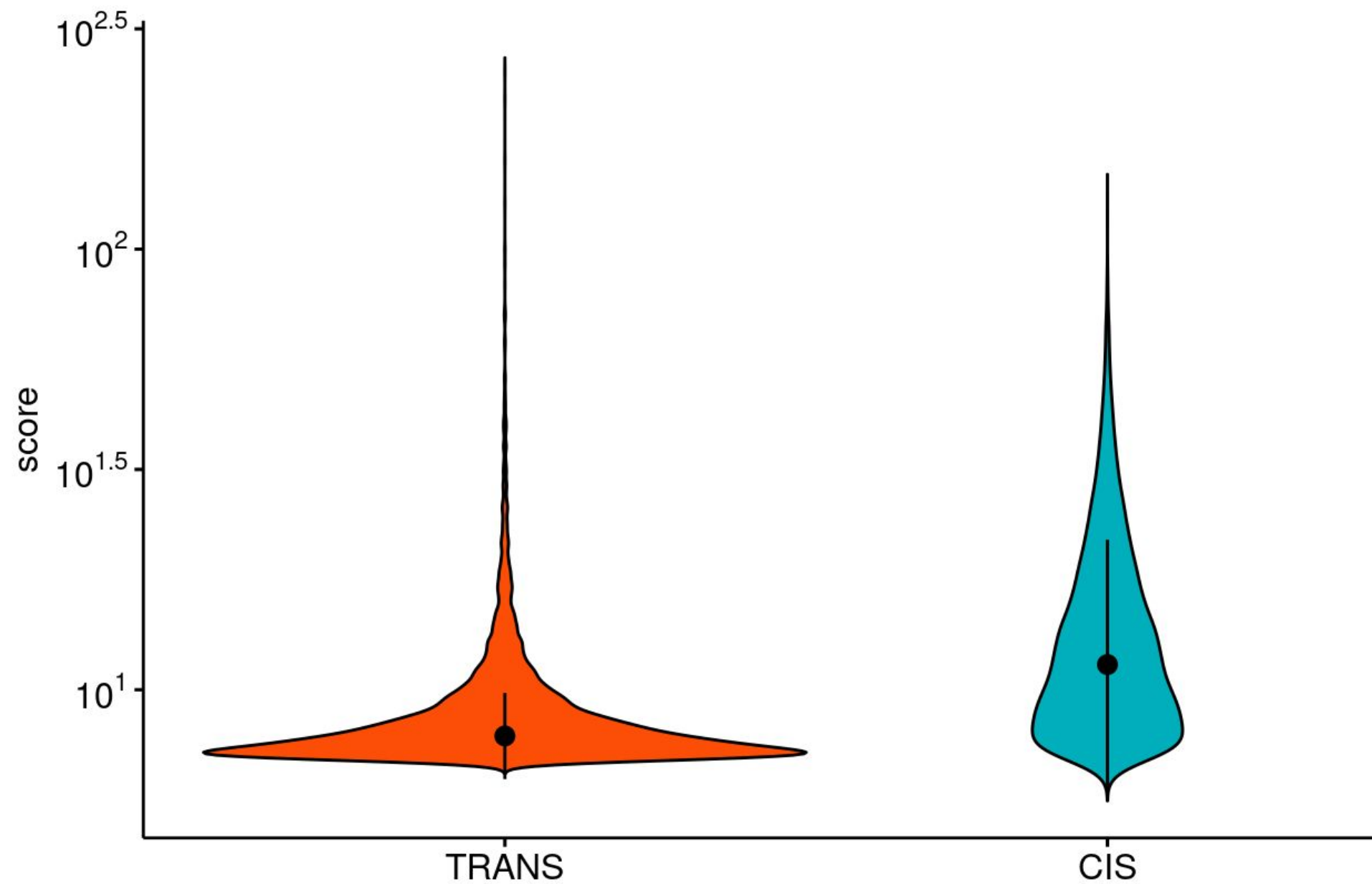

CIS TRANS

Supplement: Supplementary file 1 — Additional file 1: FigureS1. Proportion and score of cis- and trans-eQTLs. Proportions(A) and scores (B) of cis- and trans-eQTLs detectedwith the multi-locus approach. Scores correspond to the -log10 ofthe p-value of the test. [file 12864_2022_8690_MOESM1_ESM.pdf]

A)

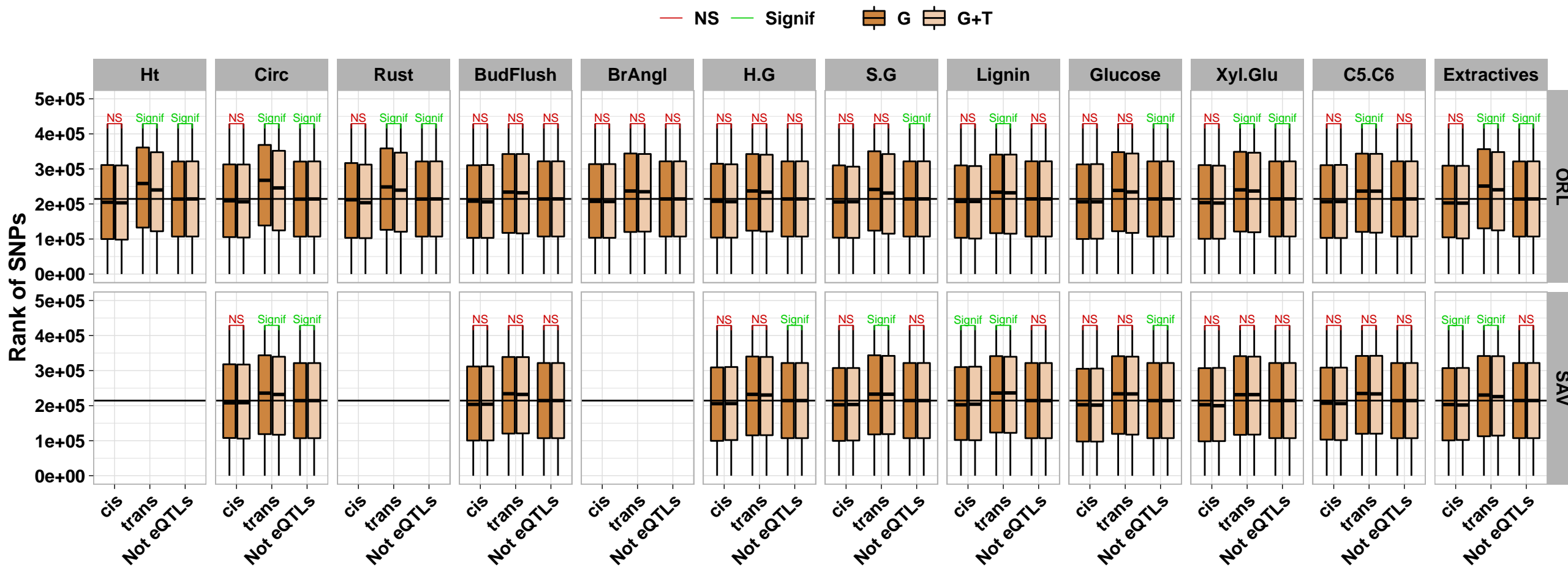

B)

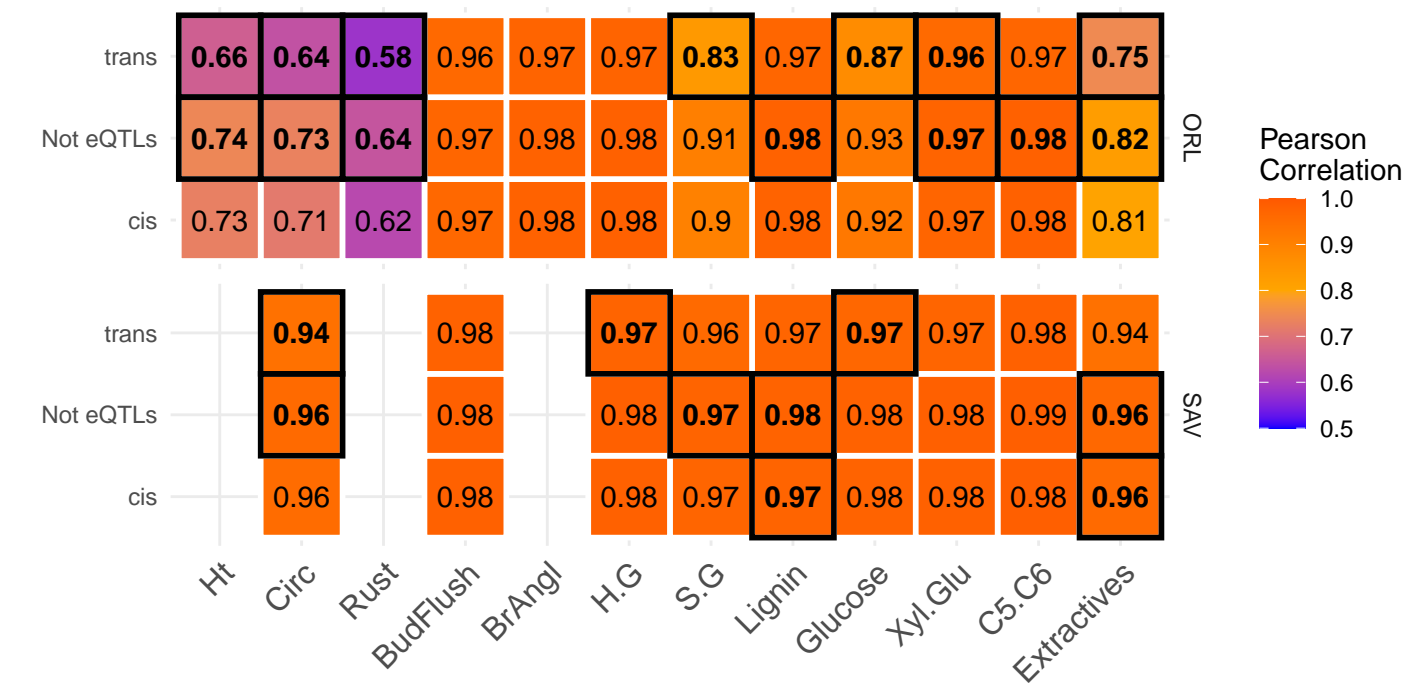

C)

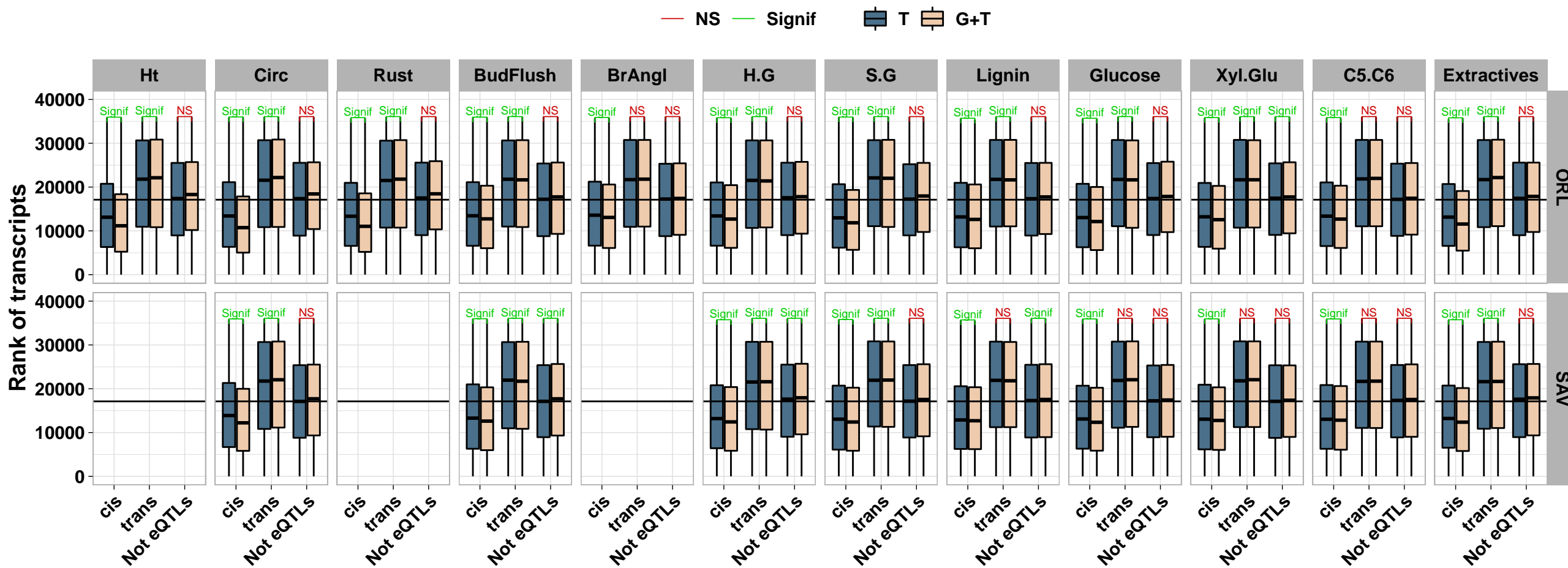

D)

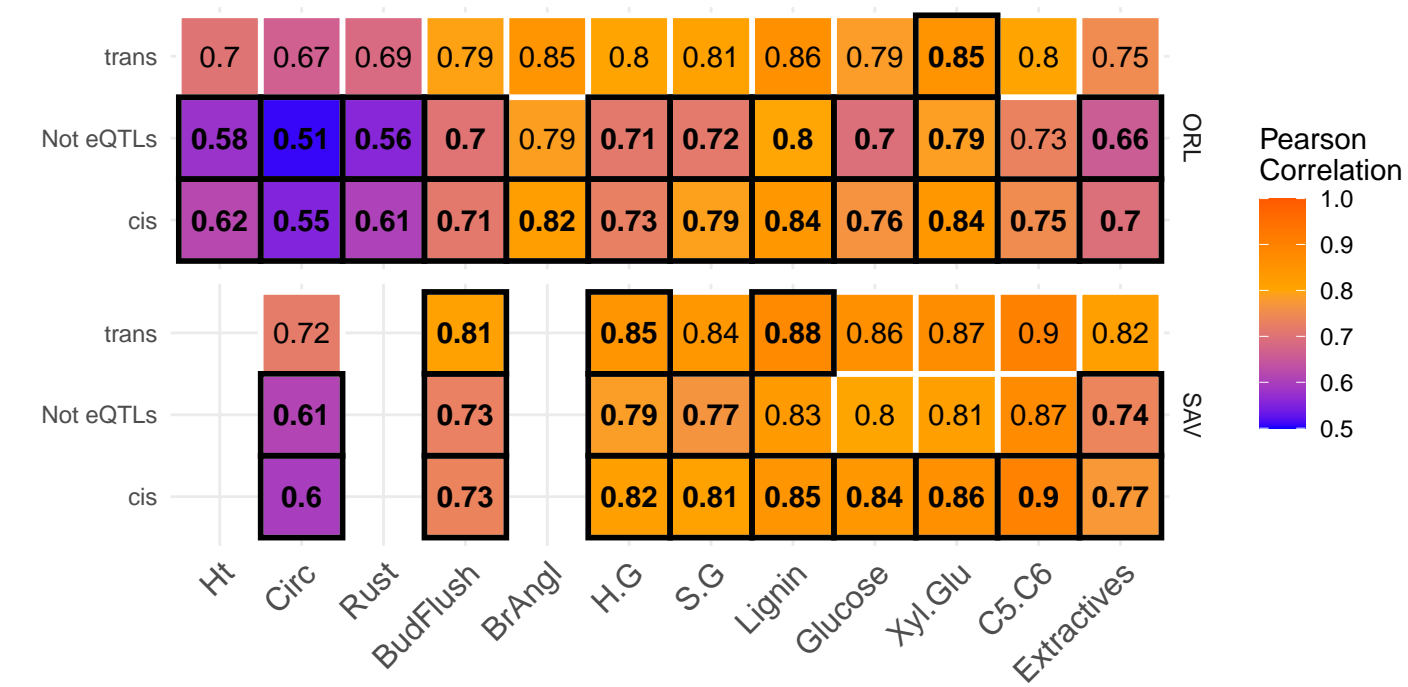

Supplement: Supplementary file 2 — Additional file 2: Figure S2. Comparison between the rank of predictors across single- and multi-omic models. Panels A and C represent the boxplots of SNPs and transcripts rank, according to the traits and sites. Features are grouped into the following categories determined after the eQTL analysis: cis or trans eQTL or regulated transcripts and not eQTLs. Panels B (SNPs) and C (transcripts) represent the correlations between the ranks of predictors across single- and multi-omic models, splitting the predictors into the same previous categories, determined from the eQTL analysis. Ranks were computed from squared effects of features in the ridge-regression models. [file 12864_2022_8690_MOESM2_ESM.pdf]

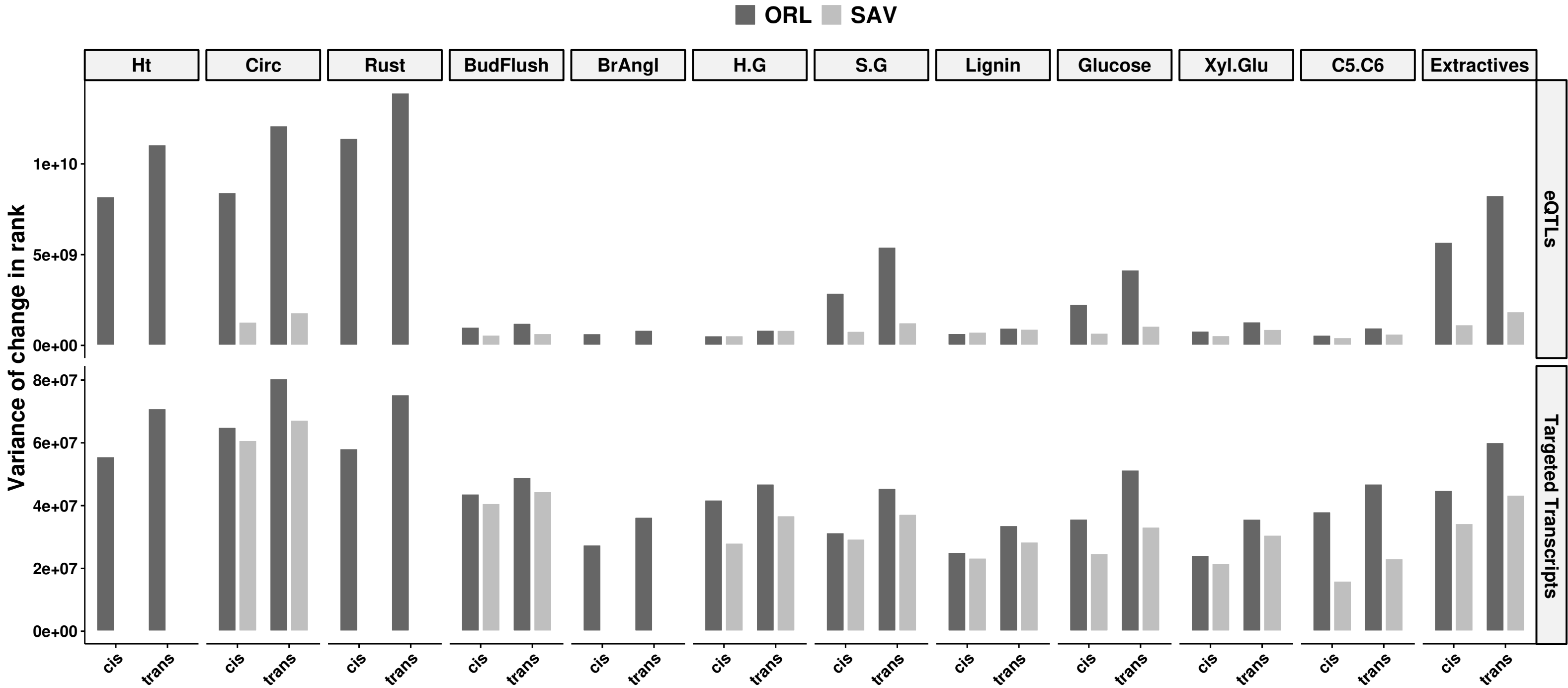

Supplement: Supplementary file 3 — Additional file 3: Figure S3. Variation of the change in rank of the eQTLs and targeted transcripts. Barplot of the variance of change in rank of eQTLs (top) and targeted transcripts (bottom) for each trait (panel) and site (dark grey: Orleans ; light grey: Savigliano). Ranks were computed from squared effects of features in the ridge-regression models. [file 12864_2022_8690_MOESM3_ESM.pdf]

trans cis Not eQTLs • Non signif \* Signif

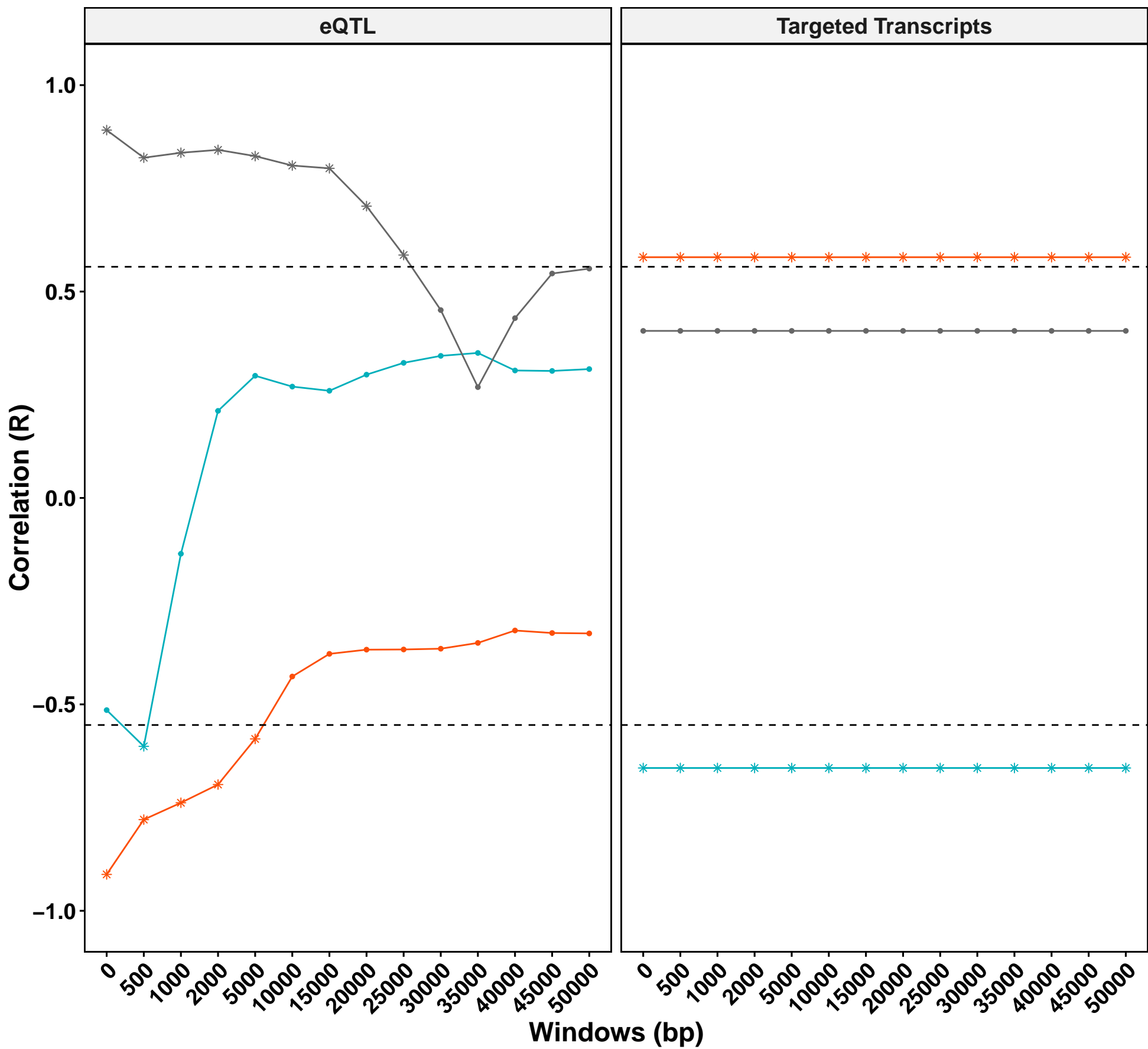

Supplement: Supplementary file 5 — Additional file 5: Figure S5. Stability of the relationships between change in predictorrank and muti-omic prediction advantage for traits measured at Orleans. The relationship is measured as the correlation coefficient between change in rank and relative advantage in prediction with the multi-omic model over the single-omic counterpart. The stability of the relationship is evaluated with respect to the categorization of eQTLs into cis, trans, or not eQTLs, being defined according to windows of increasing size in bp around the SNPs detected with the multi-locus model. [file 12864_2022_8690_MOESM5_ESM.pdf]

A) eQTLs

mean. $R^2$ (G+T) – mean. $R^2$ (G)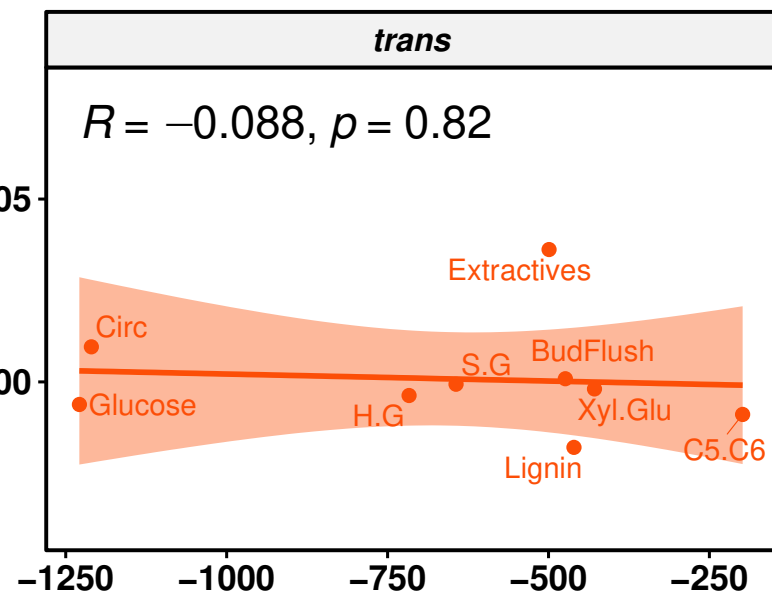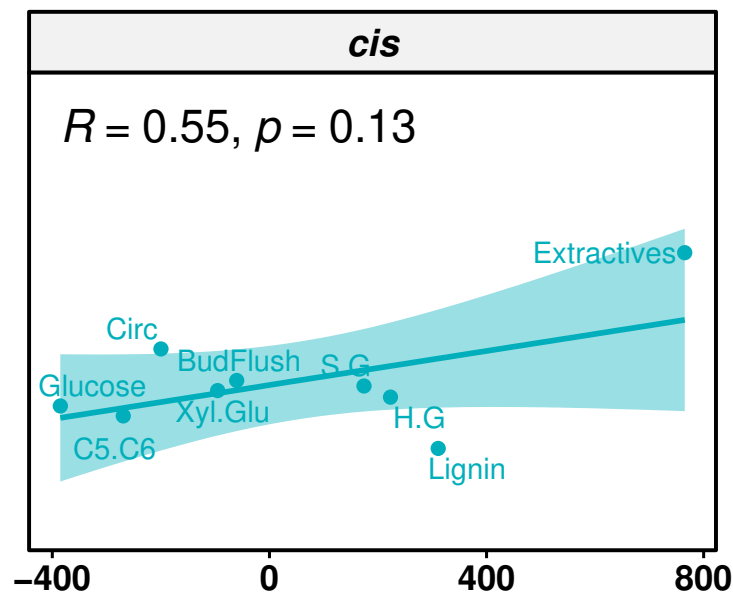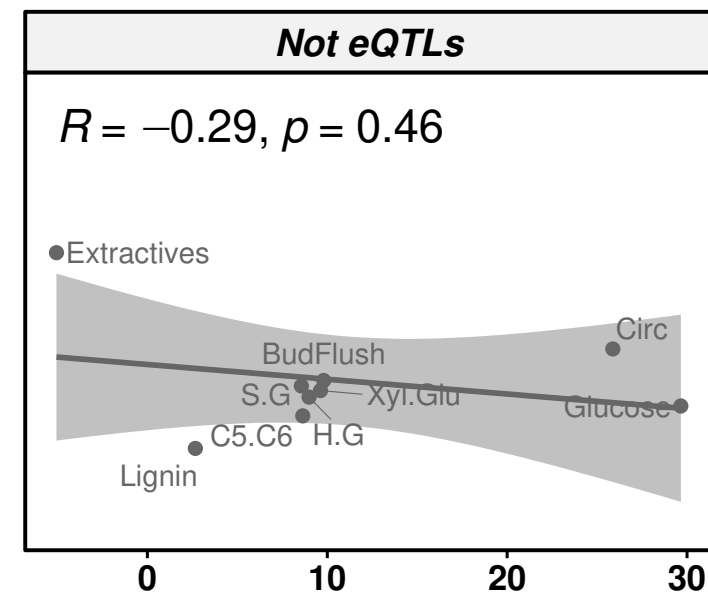

B) Targeted transcripts

mean. $R^2$ (G+T) – mean. $R^2$ (T)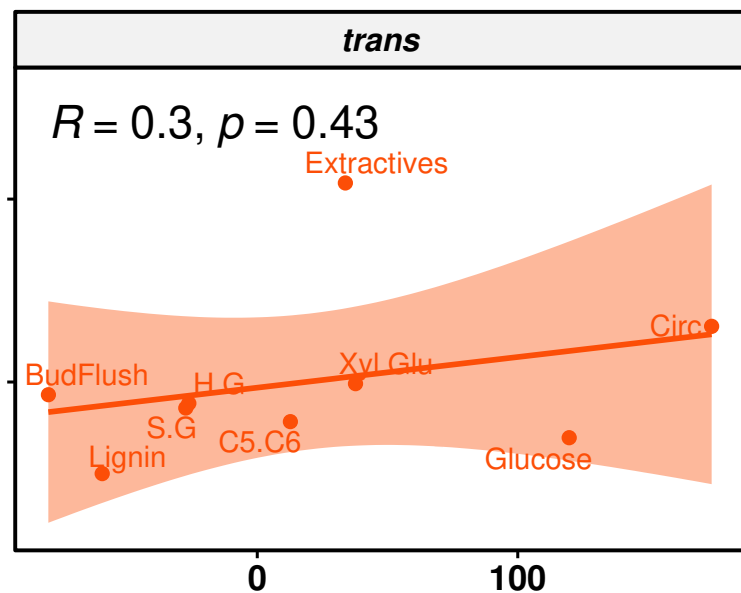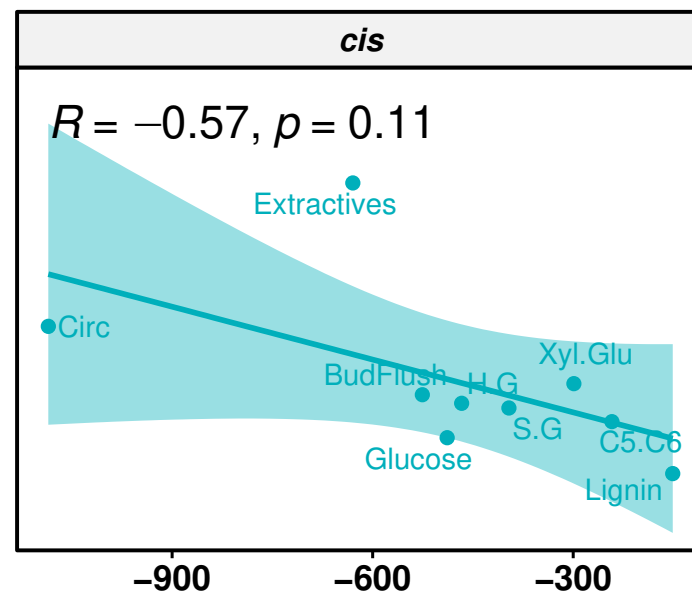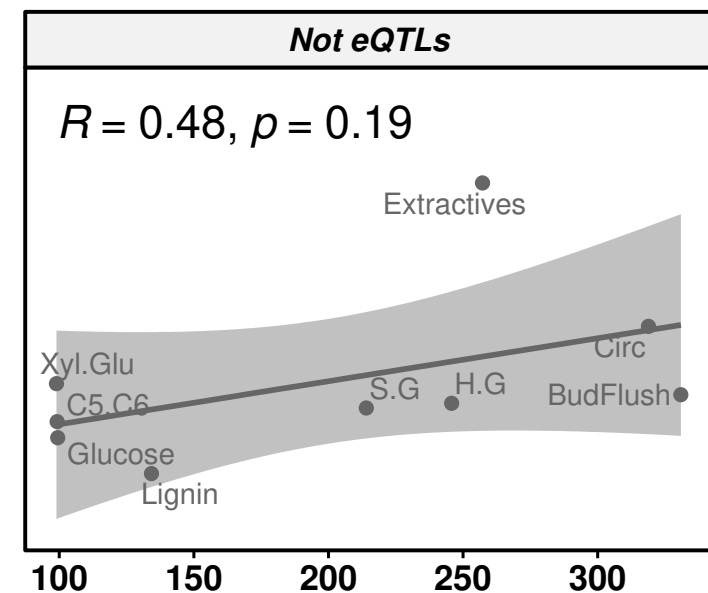

Average change in rank

Supplement: Supplementary file 6 — Additional file 6: Figure S6. Relationship between change in predictor rankand muti-omic prediction advantage for traits measured at Savigliano. Regression across traitsmeasured at Savigliano between average change in predictor rank and advantage in performance of the multi-omic model (G+T) over the single-omic counterpart. Ranks were computed from squared effects of features in the ridge-regression models. The top panel (A) shows the regression obtained with the eQTLs (trans-eQTLs on the left, cis-eQTLs in the middle, and SNPs not detected as eQTL on the right). The bottom panel (B) shows the regression obtained with the regulated transcripts (trans on the left, cis in the middle, and not found to be associated with eQTLs on the right). [file 12864_2022_8690_MOESM6_ESM.pdf]
