# Supplementary material for: eQTLs are key players in the integration of genomic and transcriptomic data for phenotype prediction
Source: BMC Genomics. 2022 Jun 28;23:476. doi: 10.1186/s12864-022-08690-7 (PMC9238188; doi:10.1186/s12864-022-08690-7)
Supplement: Supplementary file 4 — Additional file 4: Figure S4. Change in rank of the eQTLs and their corresponding targeted transcripts. Scatter plots of the changes in rank of the eQTLs and their corresponding targeted transcripts for each trait. Ranks were computed from squared effects of features in the ridge-regression models. The red and blue dots represent the regulation in transand cis, respectively. [file 12864_2022_8690_MOESM4_ESM.pdf]

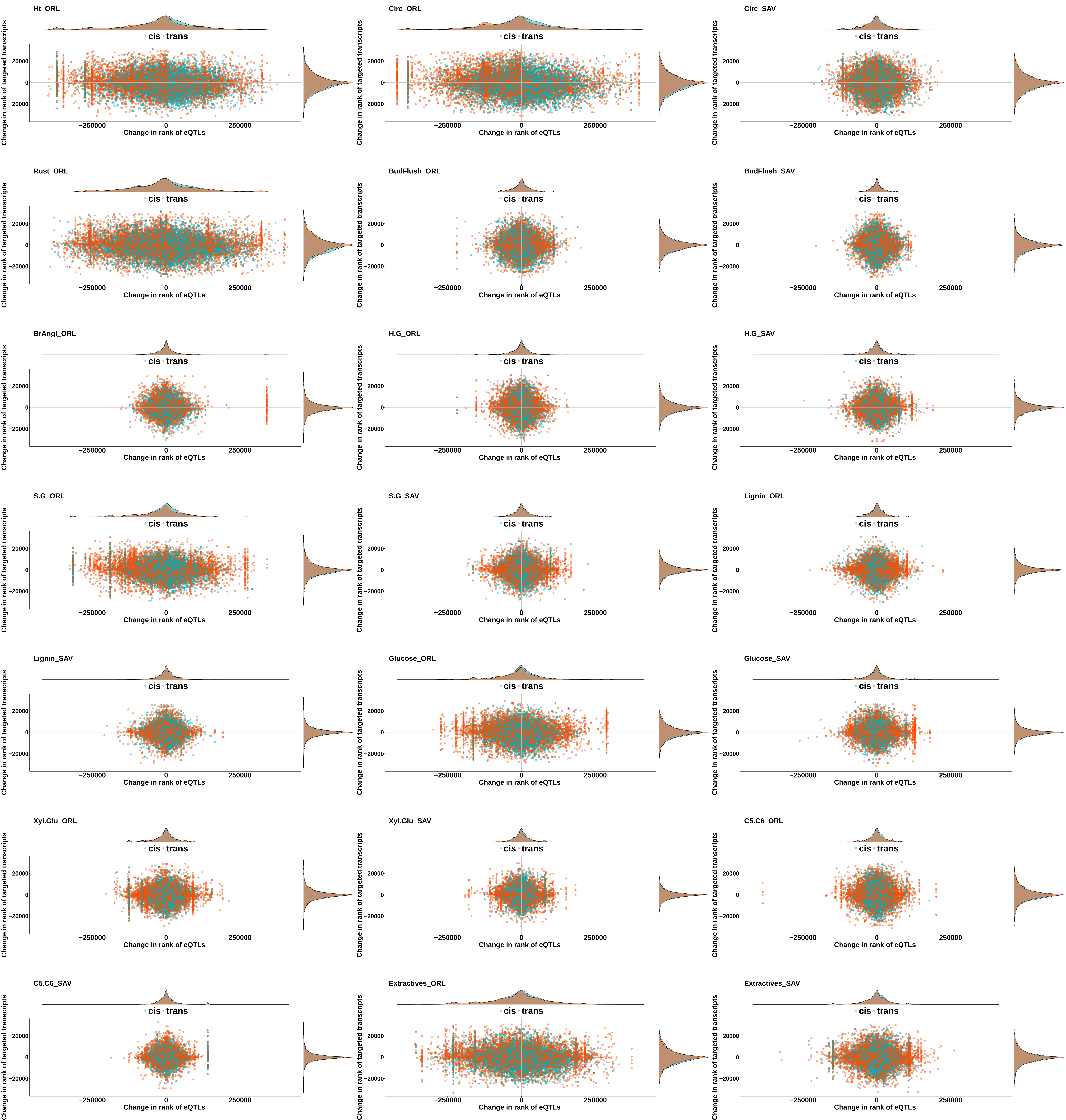

Ht_ORL
Circ_ORL
Circ_SAV
Rust_ORL
BudFlush_ORL
BudFlush_SAV
BrAngl_ORL
H.G_ORL
H.G_SAV
S.G_ORL
S.G_SAV
Lignin_ORL
Lignin_SAV
Glucose_ORL
Glucose_SAV
Xyl.Glu_ORL
Xyl.Glu_SAV
C5.C6_ORL
C5.C6_SAV
Extractives_ORL
Extractives_SAV
cis
trans
Change in rank of targeted transcripts
Change in rank of eQTLs
20000
0
−20000
−250000
0
250000
